# Reassessing Tcf21-iCre for Adult Cardiac Fibroblast Gene Deletion: Limited Recombination Efficiency Following Tamoxifen Injections

**DOI:** 10.3390/cells15151326

**Published:** 2026-07-24

**Authors:** Amelie S. Schober, Lukas Müller, Rhys Wardman, Manuela Fuhrmann, Karla Vollmerig, Felix A. Trogisch, Gergana Dobreva, Joerg Heineke

**Affiliations:** 1 Department of Cardiovascular Physiology, European Center for Angioscience (ECAS), Medical Faculty Mannheim, Heidelberg University, Ludolf-Krehl-Straße 7–11, 68167 Mannheim, Germanylukas.mueller@medma.uni-heidelberg.de (L.M.); rhys.wardman@medma.uni-heidelberg.de (R.W.); manuela.fuhrmann@medma.uni-heidelberg.de (M.F.); karla.vollmerig@medma.uni-heidelberg.de (K.V.); felix.trogisch@medma.uni-heidelberg.de (F.A.T.); 2 German Center for Cardiovascular Research (DZHK), Partner Site Heidelberg/Mannheim, 69120 Heidelberg, Germany; gergana.dobreva@medma.uni-heidelberg.de; 3 Department of Cardiovascular Genomics and Epigenomics, European Center for Angioscience (ECAS), Medical Faculty Mannheim, Heidelberg University, Ludolf-Krehl-Straße 7–11, 68167 Mannheim, Germany

**Keywords:** Tcf21, Tcf21-iCre, Cre/loxP, tamoxifen, cardiac, fibroblasts, mouse model, TAC

## Abstract

**Highlights:**

**What are the main findings?**

- Tcf21-iCre demonstrates minimal recombination efficiency in adult cardiac fibroblasts after tamoxifen injection, with mono-allelic events indicating low Cre activity.
- Neither pro-fibrotic stress nor additional tamoxifen dosing improves recombination efficiency.

**What are the implications of the main findings?**

- Tcf21-iCre cannot reliably achieve broad gene deletion in adult cardiac fibroblasts with i.p. tamoxifen protocols.
- The field requires alternative genetic tools to advance antifibrotic therapeutic strategies targeting adult fibroblasts.

**Abstract:**

Cardiac fibrosis plays a crucial role in heart failure, but research in this field remains challenging due to heterogeneity of cardiac fibroblast populations. In the past years, several Cre-drivers have been proposed for cardiac fibroblast-specific deletion of floxed genes in adult mouse research models. Here, we evaluated whether Tcf21-iCre, a tamoxifen-inducible Cre recombinase (iCre) under the endogenous Tcf21 promoter, was able to successfully target two floxed loci (*Hypoxia inducible factor 1a* (*Hif1a)* and the fluorescent reporter locus mTmG) after intraperitoneal tamoxifen injections. Six-week-old mice received intraperitoneal tamoxifen injections on five consecutive days to induce Cre activity, and the recombination efficiency was measured in isolated fibroblasts via qPCR, Western Blot and fluorescence microscopy. Recombination efficiency was very low, with minimal reduction in expression of *Hif1a*. For the mTmG locus, both bi-allelic and mono-allelic recombination was detected, with mono-allelic events suggesting low Cre activity even within *Tcf21*-expressing fibroblasts. The addition of experimental models of heart failure with reduced or preserved ejection fraction as pro-fibrotic stimuli or an inclusion of an additional round or higher doses of tamoxifen injections did not improve recombination efficiency of the targeted alleles. Thus, Tcf21-iCre targets only a small proportion of fibrogenic fibroblasts following i.p. tamoxifen injection, limiting its usability in loss-of-function studies that require broad fibroblast coverage and highlighting the need for alternative Cre-drivers or delivery strategies to facilitate antifibrotic therapy development.

## 1. Introduction

Heart failure represents a major global health challenge and has reached pandemic proportions, affecting millions of people worldwide [1]. Excessive deposition of extracellular matrix (ECM) proteins in cardiac tissue contributes to the pathogenesis of cardiac fibrosis and heart failure [2]. Tissue-resident fibroblasts synthesize and degrade ECM proteins under normal conditions to maintain tissue homeostasis. Pathological stimuli such as tissue necrosis, inflammation, or mechanical stress activate fibroblasts to proliferate or differentiate into myofibroblasts, which produce excessive ECM proteins, causing fibrosis [2,3]. There are different reasons for fibrosis, such as cardiomyocyte loss after infarction leading to replacement fibrosis or cardiac overload resulting in interstitial fibrosis. Fibrosis can be found in many myocardial diseases as well as in aging and leads to reduced cardiac function and arrhythmia because fibrotic tissue is less contractile and stiffer and changes electromechanical connection. Therefore, counteracting fibrosis would be a valuable therapeutic intervention [4].

The balance between fibroblast quiescence and activation is regulated by fibroblast intrinsic mechanisms (e.g., signaling molecules or transcription factors) as well as by cytokines and growth factors that are secreted by other cell types [2,5,6,7,8]. Because cardiac fibroblasts are an important driver of fibrosis in the adult and ageing heart [9], they have become a research focus for the development of targeted anti-fibrotic therapies.

Fibroblasts are a very heterogeneous cell population with different embryonic origins. This has made fibroblast research challenging in the past, but bulk and single-cell sequencing technologies have provided more molecular insights. As a result, several markers have been suggested for different fibroblast populations and identities [10,11].

The transcription factor Tcf21 (also known as Pod-1 or epicardin) has been identified as a marker of resident fibroblasts in the adult heart [12,13,14]. Braitsch et al. showed that resident fibroblasts express *Tcf21*, which increased upon cardiac injury [13], while Kanisicak et al. revealed *Tcf21*-expressing fibroblasts as the main source of activated fibroblasts after myocardial infarction [14].

Therefore, the expression of an inducible Cre recombinase (iCre) under the *Tcf21* promoter was suggested as an efficient technique to target resident cardiac fibroblasts in mouse research models. The Cre-loxP system is a powerful technology to precisely delete, invert or re-arrange DNA in living cells and organisms. Upon induction by tamoxifen administration, iCre translocates into the nucleus and recognizes and recombines genetically introduced loxP sites that flank a gene of interest, thereby altering its expression [15].

Our original aim was to investigate the role of hypoxia-inducible factor 1α (HIF-1α) in cardiac fibroblasts during fibrosis in models of heart failure with reduced (HFrEF) and preserved ejection fraction (HFpEF). The transcription factor HIF-1α is crucially important for the cellular adaptation to low oxygen tension. Under normoxic conditions HIF-1α protein is constantly degraded, but it accumulates rapidly during hypoxia to induce an adaptive transcriptional program to promote cell survival and oxygen delivery to tissue.

We created the Hif1aflox/flox-Tcf21-iCre mouse line, in which *Hif1a* deletion could be induced in resident cardiac fibroblasts via tamoxifen administration. To track iCre-dependent recombination, we also included the reporter locus mTmG, which indicates successful iCre activation by a switch from red (mTomato) to green (mGFP) fluorescence.

Here, we report challenges with achieving efficient gene deletion in the resident cardiac fibroblast population using Tcf21-iCre and intraperitoneal tamoxifen injections.

## 2. Materials and Methods

### 2.1. Cell Culture and Cell Isolation

#### 2.1.1. Isolation of Cardiac Fibroblasts for Cell Culture

Adult mice were euthanized by cervical dislocation, followed by intraventricular injection of 100 µL heparin (500 U/mL) into the still-beating left ventricle to prevent coagulation. Hearts were excised and placed in a Petri dish containing cold KCl solution. The atria were removed, and the ventricles were transferred to a second Petri dish with cold PBS, minced into small fragments and collected in a 15 mL Falcon tube. Ventricles of three hearts from the same genotype were pooled together to achieve sufficient cell numbers for culture.

Three milliliters of a “SADO mix” (20 mM HEPES-NaOH, 130 mM NaCl, 3 mM KCl, 1 mM NaH_2_PO_4_, 4 mM glucose and 3 mM MgSO_4_ in ddH_2_O) was added to the tissue, followed by centrifugation at 200 *g* for 3 min at 4 °C and sedimentation for an additional 3 min. The supernatant was removed and replaced with a 4 mL “SADO mix” supplemented with Liberase TH (Roche, Basel, Switzerland, Liberase TH research grade, 5401151001) in a concentration of 0.25 µg/mL. The Falcon tube was rotated at 18 rpm for 8 min at 37 °C and then allowed to sediment for 5 min. The supernatant, containing cardiac fibroblasts, was transferred into a fresh Falcon tube containing 10 mL horse serum and placed on ice to inactivate the enzyme. This digestion step was repeated twice, each time collecting the supernatant into a new tube containing 10 mL horse serum.

For the subsequent digestion, the SADO mix with the enzyme was diluted in a 1:3 dilution in the SADO mix. Five milliliters of this solution was added to the remaining tissue and rotated at 18 rpm for 25 min at 37 °C. After a 5 min sedimentation period, the supernatant was transferred to a fresh Falcon tube containing 10 mL horse serum. This procedure was repeated twice, with the final digestion performed for 15 min. The residual tissue fragments were dissociated mechanically by pipetting and transferred into a new Falcon tube containing 10 mL horse serum.

All tubes were centrifuged at 400× *g* for 3 min at 4 °C. The pellets were pooled and resuspended in 2 mL foetal calf serum (FCS) and centrifuged at 400× *g* for 3 min at 4 °C again. The pelleted cells were resuspended in DMEM supplemented with 10% FCS and seeded in a 10 cm culture dish. Cells were incubated at 37 °C in a humidified atmosphere containing 5% CO_2_. After 3 h, the media was replaced following a PBS wash to remove non-adherent cells. Confluent fibroblast cultures were subsequently passaged and used for downstream experiments.

#### 2.1.2. Isolation of Cardiac Fibroblasts via Magnetic Activated Cell Sorting

Adult mice were euthanized by cervical dislocation, hearts were excised, and surrounding fat and lung tissue were removed before washing in phosphate-buffered saline (PBS). Blood clots were removed with fine tweezers. Hearts were then transferred into dishes containing 1 mL of enzyme solution per heart. The enzyme solution was freshly prepared by dissolving 500 U/mL collagenase type I (Worthington, CatNo LS004196) and 150 U/mL DNase I (Worthington, Lakewood, NJ, USA, CatNo LS002139) in RPMI 1640 medium without glutamine (Thermo Fisher, Gibco, Waltham, MA, USA, Ref 31870025) and filtering through a 0.2 μm syringe filter.

Atria and aorta were removed, and hearts were cut into small pieces, which were transferred into 15 mL Falcon tubes containing 4 mL enzyme solution. The samples were incubated at 37 °C on a cell culture rotator set to 16–18 rpm for 45 min to allow enzymatic digestion.

Upon completion of digestion, the solution was filtered through 70 μm nylon cell strainers, and 5 mL warm FCS was added to inhibit enzyme activity. The filters were washed with 10 mL MACS buffer, which was prepared by adding 2.5 mL MACS BSA stock solution (Miltenyi, Bergisch Gladbach, Germany, Ref 130-091-376) to 500 mL MACS rinsing solution (Miltenyi, Ref 130-091-222). Cell suspensions were centrifuged at 400× *g* for 6 min at 4 °C. Pellets were washed with 10 mL MACS buffer and centrifuged again.

Endothelial cells were isolated by resuspending cell pellets in 500 μL MACS buffer and incubating with 20 μL CD146 microbeads (Miltenyi, 130-092-007) on a rotator at room temperature for 30 min. After washing with 10 mL MACS buffer, cells were centrifuged again. Magnetic MS columns (Miltenyi, Ref 130-042-201) were primed with 500 μL MACS buffer on a magnetic stand, then cell suspensions were loaded onto the columns, and the flow-through containing non-endothelial cells was collected in 15 mL tubes on ice. Columns were washed three times with 500 μL MACS buffer. Endothelial cells that adhered to the columns were eluted by removing the column from the magnet and pushing approximately 2 mL MACS buffer through the column with a piston into new collection tubes. The eluates were centrifuged at 400× *g* for 6 min at 4 °C, and supernatants were discarded. Cell pellets were lysed with RA1 buffer containing β-mercaptoethanol for gene expression analysis.

The flow-through was centrifuged, and pellets were incubated with 20 μL feeder removal microbeads (Miltenyi, 130-095-531) for positive selection of fibroblasts for 30–40 min on a rotator at room temperature, then washed and centrifuged. Magnetic MS columns were primed with MACS buffer on a magnetic stand before cell suspensions were loaded. Bound fibroblasts were washed three times. An additional purification step was performed by loading the eluate directly onto a second primed MS column, which was subsequently washed three times. Elution of fibroblasts was performed by pushing approximately 2 mL MACS buffer through the column with a piston into fresh tubes. Eluates were centrifuged at 400× *g* for 6 min at 4 °C. Pellets were lysed with RA1 buffer plus β-mercaptoethanol for gene expression analysis. The purity of cFb in this method is >90%, as shown in previous works [16].

#### 2.1.3. In Vitro Cell Culture Treatments with 4-Hydroxy-Tamoxifen

Cells were treated with the active tamoxifen metabolite 4-Hydroxy-Tamoxifen at a concentration of 500 nM for three days.

#### 2.1.4. In Vitro Cell Culture Treatments with Dimethyloxalylglycine

Cells were incubated with 1 mM DMOG in DMSO for four hours and lysed thereafter.

#### 2.1.5. Viruses

For expression studies in Tcf21-iCre- isolated cultured cardiac fibroblasts, cells were infected with a replication-deficient adenovirus-encoding Cre recombinase (Ad-Cre) with a multiplicity of infection of 50 to induce loxP site recombination. As a control, a replication-deficient adenovirus-encoding β-galactosidase (Ad-β-Gal) with a multiplicity of infection of 50 was used. Both viruses have been tested and used before by Molkentin et al. [8]. Cells were incubated with the virus for 4 h, washed and analyzed five days later.

### 2.2. Animal Models

#### 2.2.1. Mouse Line

Mice expressing tamoxifen-inducible Cre recombinase (iCre) under the control of one allele of the endogenous *Tcf21* promoter (MGI ID: MGI:5442346) [17] were crossed with homozygous floxed *Hif1a* mice (MGI ID: MGI:3711123) to obtain a knockout of *Hif1a* specifically in *Tcf21*-expressing fibroblasts after tamoxifen-dependent Cre induction. In the Tcf21-iCre-mice exon 1 of *Tcf21* was replaced with a MerCreMer cassette. These mice were crossed with homozygous mTmG reporter mice (MGI ID: MGI:3716464), resulting in the genotype C57BL/6 Hif1atm3Rsjo Tcf21tm3.1(cre/Esr1*)Eno Gt (ROSA)26Sortm4 (ACTB-tdTomato-EGFP)Luo.

Mice were assigned to experimental groups based on genotyping results (Tcf21-iCre- or Tcf21-iCre+) and were subsequently randomized to different treatment conditions. The investigators conducting the experiments were blinded to the genotypes of the mice. Animals had unrestricted access to food and water throughout the study.

#### 2.2.2. Gene Knockout Induction

Mice were injected intraperitoneally with tamoxifen for five consecutive days to activate iCre recombinase. Six-week-old mice received 100 µL of tamoxifen (Sigma-Aldrich, St. Louis, MO, USA, T5648; 5 mg/mL, dissolved in miglyol) per 20 g of body weight per day, corresponding to a total daily dose of 25 mg of tamoxifen per kilogram of body weight. In mice subjected to two rounds of induction, the same injection regimen was repeated at eight weeks of age. This tamoxifen regimen is established in our group and showed good gene knockout induction [18]. A total of 75 mg/kg bodyweight tamoxifen/day was administered to Tcf21-iCre mT/mG reporter mice in a similar manner, but with the injection of 15 mg/mL tamoxifen in miglyol in solution.

#### 2.2.3. Mouse Genotyping

Tissue from ear markings was genotyped using the following primers: geno_Tcf21 endogenous forward (5′-TTCTCCAGGCTCAAGACCAC-3′), Tcf21iCre_forward (5′-GCTTCCGATATCCAGATCCAGAC-3′), Tcf21 endogenous reverse (5′-CAAACCCTAGCACAAATCACTCGC-3′), mTmG mouse forward (5′-CTCTGCTGCCTCCTGGCTTCT-3′), mTmG mouse wildtype reverse (5′-CGAGGCGGATCACAAGCAATA-3′), mTmG mouse mutant reverse (5′-TCAATGGGCGGGGGTCGTT-3′), Hif1a mouse forward (5′-GCAGTTAAGAGCACTAGTTG-3′) and Hif1a mouse reverse (5′-GGAGCTATCTCTCTAGACC-3′).

#### 2.2.4. Transverse Aortic Constriction

Analgesia was administered 20–30 min before surgery via subcutaneous injection of buprenorphine (0.1 mg/kg body weight) and Rimadyl (carprofen, 5 mg/kg). Anesthesia was induced in an incubation chamber with 3% isoflurane at an airflow rate of 1 L/min. During the surgical procedure, mice were placed on a heated operating table maintained at 37 °C, and anesthesia was maintained by mask inhalation with 3% isoflurane.

A median skin incision was made from the level of the fourth rib until the cartilage of the trachea became visible, corresponding to approximately 2–3 cm. Following careful dissection of the musculoskeletal thoracic wall and trachea, endotracheal intubation was performed orally under direct visual control. Controlled ventilation was initiated using a Harvard respirator (Harvard Apparatus, Holliston, MA, USA, model 683), with anesthesia maintained by 2% isoflurane inhalation.

After non-invasive fixation of the animal on the operating platform, a partial median sternotomy was performed up to the level of the third rib (approximately 5 mm). The thymus was gently separated to expose the aortic arch. The aortic arch was then ligated between the origins of the two carotid arteries using surgical suture material (Ethicon, Raritan, NJ, USA, Ethilon 7-0) placed around a 26-gauge needle, which was immediately removed after ligation. To prevent bradyarrhythmias, atropine sulphate (0.1 mg/kg body weight, subcutaneously) was administered once during the procedure.

Following ligation, the sternum was realigned, and the thoracic musculature was closed using interrupted sutures. The skin was closed with sutures (Ethicon, Ethibond Excel 5-0) and sealed with tissue adhesive (B. Braun, Melsungen, Germany, 1050052). Isoflurane administration was then discontinued, and ventilation was continued with pure oxygen. The return of spontaneous respiration was monitored by observing accessory respiratory muscle activity or by transient disconnection of the endotracheal tube from the ventilator. Once spontaneous breathing resumed, the animal was extubated only after restoration of reflex responses. Each animal was kept in a temperature-controlled incubator set at 28 °C until fully awake. The total duration of the surgical procedure was approximately 15 min.

Sham-operated animals underwent the same procedure except that no aortic ligation was performed. Postoperative analgesia consisted of subcutaneous buprenorphine administration (0.1 mg/kg, three times daily at 6–8 h intervals) for the first three postoperative days and metamizole supplied in the drinking water (200 mg/kg body weight) for seven days postoperatively.

#### 2.2.5. Induction of Heart Failure with Preserved Ejection Fraction Phenotype

We used the model established by Schiattarella et al. [19] that consists of the combination of a high-fat diet with 60% of the energy as fat (Brogaarden, Kopenhagen, Denmark, D12492) and the nitric oxide synthase inhibitor N(ω)-nitro-L-arginine methyl ester (L-NAME, Sigma Aldrich, St. Louis, Missouri, N5751-25) in a concentration of 0.5 g/L in the drinking water over ten weeks.

### 2.3. Analysis

#### 2.3.1. qPCR

RNA was purified using the NucleoSpin RNA kit by Macherey-Nagel (Macherey Nagel, Düren, Germany, 740955.250) as described in the company’s protocol. The synthesis of cDNA was performed using the Maxima H Minus First Strand cDNA Synthesis Kit by Thermo Scientific (Thermo Scientific, Waltham, MA, USA, K1651).

The qPCR was performed using 10 ng of cDNA and a primer concentration of 500 nM. Hypoxanthine-Guanine Phosphoribosyl transferase (HPRT) was used as a normalizer of relative cDNA content. The primers used were Hif1a mouse forward (5′-CATCAGTTGCCACTTCCCCA-3′), Hif1a mouse reverse (5′-GGCATCCAGAAGTTTTCTCACAC-3′), Hprt mouse forward (5′-AAGCTTGCTGGTGAAAAGGA-3′), Hprt mouse reverse (5′-TTGCGCTCATCTTAGGCTTT-3′), dTomato mouse forward (5′-ACCTGTTCCTGGGGCATG), dTomato mouse reverse (CCATGTTGTTGTCCTCGGAG-3′), Tcf21 mouse forward (5′-CCAGTCAACCTGACGTGGC-3‘), Tcf21 mouse reverse (5′-CAGAGCTCACGGTTCCCAG-3‘), Pecam1 (CD31) mouse forward (5′-CACTCCGGGAAGTACAAATGCACA-3′), Pecam1 (CD31) mouse reverse (5′-GCAAGGAACAATTGACCGTCACGA-3′), PDGFRa mouse forward (5′-CTGCCAGACATTGACCCTGT-3′), PDGFRa mouse reverse (5′-GAACCTGTCTCGATGGCACT-3′), TNNT2 mouse forward (5′-CCTGGAGGCTGAGAAGTTCG-3′) and TNNT2 mouse reverse (5′-TTGGCCTTCCCACGAGTTTT-3′).

#### 2.3.2. Western Blot

Samples were lysed in 1.5× Laemmli buffer and then sonicated. Before analysis, samples were boiled at 95 °C for 15 min. Concentration was quantified using the Pierce™ BCA Protein Assay Kit (Thermo Scientific, 23225).

Between 25 and 40 µg of protein together with 5% beta-mercaptoethanol and 5% of bromphenol blue was loaded onto a 7.5% or 10% polyacrylamide gel, along with the corresponding protein marker for reference. Gel electrophoresis was performed at 100 V.

Proteins were transferred onto a PVDF membrane at 100 V for one hour. Following the transfer, the membrane was stained with Ponceau solution, photographed and destained by washing with double-distilled water. The membrane was then blocked for one hour in 5% milk powder dissolved in TBST.

After blocking, the membrane was incubated overnight at 4 °C with primary antibodies diluted in 5% milk in TBST. The following day, the membrane was washed three times for 10 min each in TBST. The following antibodies were used for Western blotting in the indicated dilution: HIF1α (1:000, Novus Biologicals, Centennial, CO, USA, NB100-479), β-actin (1:1000, Abcam, Cambridge, England, ab115777) and GFP (1:1000, Roche, 11814460001).

Next, the membrane was incubated with horseradish peroxidase-conjugated secondary antibodies anti-rabbit HRP (Amersham, Little Chalfont, England, NA934) or anti-mouse HRP (Thermo Fisher, 62-6520) diluted 1:10,000 in 5% milk in TBST for one hour. After three washes for 10 min each with TBST and incubation with ECL, chemiluminescence was recorded on an Amersham Imager.

#### 2.3.3. Immunohistochemistry

Embedded tissue samples were sectioned using a rotary cryotome (Thermo Fisher, HM35S) at a thickness of 7 µm. Sections were air-dried at room temperature for 30 min and subsequently stored at −20 °C until further use.

Slides were fixed in 4% paraformaldehyde solution (Rotihistofix, Carl Roth, Karlsruhe, Germany, P087.1) for 15 min at room temperature. Slides were then washed three times for 5 min in PBS. Permeabilization was performed with 0.3% Triton X-100 in PBS for 20 min, followed by three additional 5 min washes in PBS. Samples were blocked with 3% BSA in PBS for 30 min at room temperature. Primary antibody incubation was carried out overnight at 4 °C in a dark, humid chamber.

For PDGFRa staining, the anti-PDGFRα antibody (goat, R&D, Minneapolis, MN, USA, AF1062) was used at a dilution of 1:70. As a negative control, 3% BSA in PBS was applied instead of the primary antibody. As secondary antibody Alexa Fluor 633 (Invitrogen, Carlsbad, CA, USA, A-21082) or Alexa Fluor 405 (donkey, Invitrogen, A-48529) was applied at a dilution of 1:200 in 3%BSA in PBS and incubated for 3 h at room temperature in a dark, humid chamber. After incubation, slides were washed three times for 5 min in PBS, mounted with Rotimount Fluocare (Carl Roth, HP19.1) and allowed to dry overnight. Slides were then stored at 4 °C until imaging.

For the PDGFRa and Cre co-staining, slides were processed the same way. The antibodies used were anti-Cre antibody 1:100 (rabbit, GeneTexGTX127270) and anti-PDGFRα antibody (goat, R&D AF1062) 1:100. Cre subcellular localization was analyzed by using a rabbit anti-Cre antibody (Abcam, ab190177) at a dilution of 1:50. The following day, slides were washed three times for 5 min in PBS. The secondary antibodies used were anti-goat IgG Alexa Fluor 633 (donkey, Life Technologies, Carlsbad, CA, USA, A-21082) and anti-rabbit Alexa Fluor 405 (goat, Invitrogen, A48254) or anti-goat AF488 (donkey, Life Technologies, A-11055) at 1:200. They were mounted with Rotimount Fluocare (Carl Roth, HP19.1) or with mounting medium with DAPI (Santa Cruz Biotechnology, Dallas, TX, USA, sc-24941).

Isolated cardiac fibroblasts in cell culture were washed several times with PBS, then fixed with 4% PFA in PBS, washed again with PBS and mounted with Rotimount Fluocare (Carl Roth, HP19.1). Images of living cell cultures were taken on a Leica DMi8 (Leica Microsystems, Wetzlar, Germany) inverted microscope. Fixed cells and tissue images were taken on a Zeiss LSM 800 microscope (Carl Zeiss, Jena, Germany).

### 2.4. Statistics

Statistical analyses were performed using GraphPad Prism software (version 5.01). Depending on the experimental design, either Student’s *t*-test or one-way ANOVA with Brown–Forsythe and Welch correction was applied. A *p*-value <0.05 was considered statistically significant.

## 3. Results

**Figure 1:**
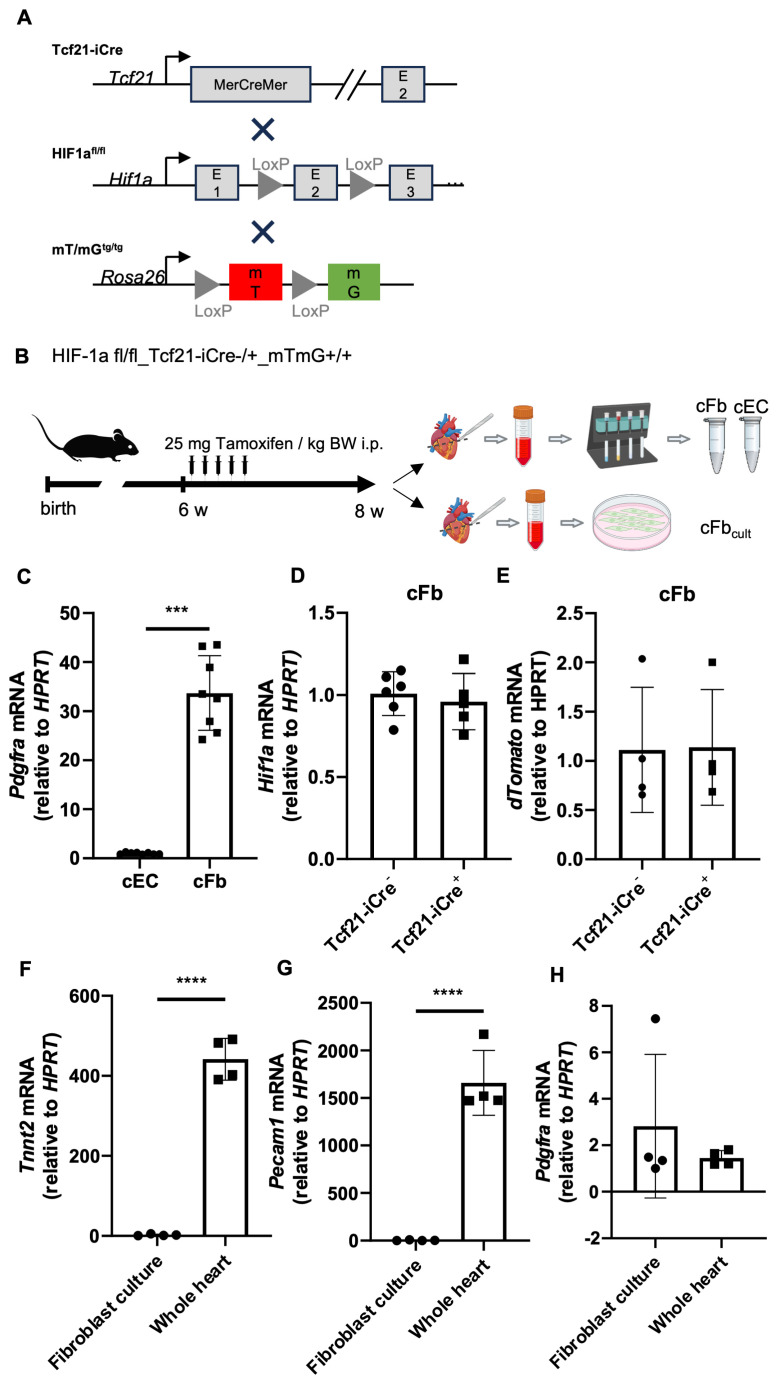

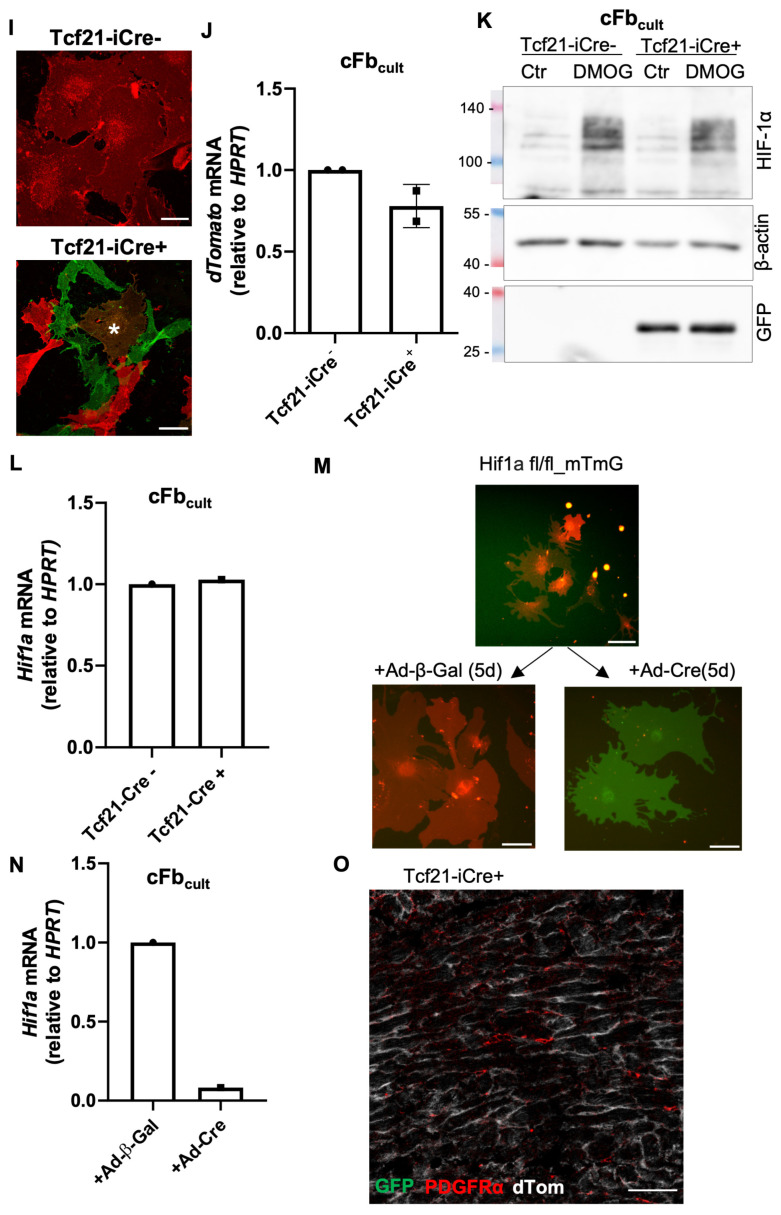
Only a subset of resident cFb are targeted upon i.p. tamoxifen delivery under homeostatic conditions in Tcf21-iCre+ mT/mG mice. (**A**) Mouse breeding scheme. E: exon; mT: mTomato; mG: mGFP (**B**) Experimental scheme: two weeks after the tamoxifen application, ventricular fibroblasts were isolated via MACS or isolated and cultured. (**C**) MACS-sorted cFb and cEC were lysed, mRNA was extracted, and *PDGFRα* mRNA was measured via qPCR; *** *p* ≤ 0.0001. (**D**) *Hif1a* mRNA was measured via qPCR in MACS-sorted cFb from Tcf21-iCre- and Tcf21-iCre+ mice. (**E**) *dTomato* mRNA was measured via qPCR in MACS-sorted cFb from Tcf21-iCre− and Tcf21-iCre+ mice. (**F**) *Tnnt2* mRNA was measured via qPCR in cultured fibroblasts Tcf21-iCre−/+ and in the whole heart from Tcf21-iCre−/+ mice; **** *p* ≤ 0.00001. (**G**) *Pecam1* mRNA was measured via qPCR in cultured fibroblasts Tcf21-iCre−/+ and in the whole heart from Tcf21-iCre−/+ mice; **** *p* ≤ 0.00001. (**H**) *Pdgfra* mRNA was measured via qPCR in cultured fibroblasts Tcf21-iCre−/+ and in the whole heart from Tcf21-iCre−/+ mice. (**I**) cFb were cultured and imaged live (scale bar: 50 μm). * Indicates a dTomato/GFP double-positive cell. (**J**) *dTomato* mRNA was measured via qPCR in cFb_cult_. For each data point, ventricles of at least three individual mice with the same genotype were pooled. (**K**) cFb_cult_ were incubated with 1 mM DMOG or vehicle for 4 h, lysed, separated by SDS-PAGE and immunoblotted with anti-HIF-1α, anti-GFP and anti-β-actin antibodies. For each data point, ventricles of at least three individual mice with the same genotype were pooled. (**L**) *Hif1a* mRNA was measured via qPCR in cFb_cult_. For each data point, ventricles of at least three individual mice with the same genotype were pooled. (**M**) cFb_cult_ from Tcf21-iCre^−^ mice without tamoxifen treatment were transduced with adenoviruses carrying β-galactosidase (Ad-β-Gal) or Cre recombinase (Ad-Cre) and imaged live 5 d later (scale bar: 50 μm). For each data point, ventricles of at least three individual mice with the same genotype were pooled. (**N**) *Hif1a* mRNA was measured via qPCR in cFb_cult_. For each data point, ventricles of at least three individual mice with the same genotype were pooled. (**O**) Tcf21-iCre^+^ mice were treated with tamoxifen (5 d i.p., 25 mg/kg BW/day), and two weeks later, hearts were extracted, embedded, sectioned and stained for PDGFRα and endogenous GFP (scale bar: 50 μm).

To deplete HIF-1α from adult cardiac fibroblasts, we generated the Hif1a-fl/fl-Tcf21-iCre mouse line. The iCre recombinase is expressed from the *Tcf21* genomic locus under the control of the endogenous *Tcf21* promoter and should recombine the genomic *Hif1a* locus in cardiac fibroblasts in the presence of tamoxifen [17]. For efficient monitoring of Cre-dependent genomic recombination, we crossed the Hif1a-fl/fl-Tcf21-iCre mouse line with the mTmG reporter line, which shows red fluorescence (dTomato) prior and green fluorescence (GFP) post Cre-mediated recombination (Figure 1A).

### 3.1. A Subset of Cardiac Fibroblasts Is Targeted with Tcf21-iCre

Intraperitoneal tamoxifen (at 25 mg/kg body weight/day) was administered at 5 consecutive days to 6-week-old Hif1a-fl/fl-Tcf21-iCre^+^mTmG (Tcf21-iCre+) and Hif1a-fl/fl-Tcf21-iCre^—^mTmG (Tcf21-iCre-) mice to induce Cre-mediated recombination at the *Hif1a* and mTmG genomic loci (Figure 1B). Two weeks later, mice were euthanized, and cardiac fibroblasts (cFb) and cardiac endothelial cells (cEC) were isolated from ventricular tissue by magnetic-activated cell sorting (MACS) and immediately lysed for RNA extraction. This isolation protocol is known to yield high-purity cFb populations [16]; accordingly, mRNA expression of the fibroblast marker *Pdgfra* was significantly enriched in cFb compared to cEC (Figure 1C). However, *Hif1a* mRNA levels in cFb from Tcf21-iCre+ mice were not significantly reduced compared to Tcf21-iCre- controls (Figure 1D), suggesting a low recombination efficiency of the *Hif1a* locus. Similarly, dTomato mRNA levels did not decrease in cFb from Tcf21-iCre+ animals compared with controls (Figure 1E), suggesting inefficient recombination of the mTmG locus as well.

To get further insights into the inefficient targeting at the cellular level, we isolated cFb from ventricular tissue two weeks post-tamoxifen injection and cultured them (cFb_cult_). We performed qPCR to control the purity of the fibroblast culture. We detected a strong reduction in *Tnnt2* and *Pecam1* mRNA in cultured mouse fibroblasts versus whole hearts, indicating de-enrichment of cardiomyocytes and endothelial cells, while *Pdgfra* expression was maintained, indicating high fibroblast content (Figure 1F–H).

As expected, all cFb_cult_ derived from Tcf21-iCre- mice showed exclusively red fluorescence. A subset of the cFb_cult_ from Tcf21-iCre+ mice displayed green fluorescence (Figure 1I), which indicated that at the time of tamoxifen administration, iCre was expressed in some of the cells. We also observed cFb_cult_ exhibiting mixed red and green fluorescence (Figure 1I, *), implying recombination of only one allele of the mTmG locus. In line with this, dTomato mRNA levels were only slightly reduced in cFb_cult_ from Tcf21-iCre^+^ mice (Figure 1J).

To analyze the recombination efficiency of the *Hif1a* locus, we treated cFb_cult_ with a hypoxia mimetic drug (DMOG) for 4 h to protect HIF-1α protein from oxygen-dependent degradation. HIF-1α protein levels were comparable in cFb_cult_ from both Tcf21-iCre- and Tcf21-iCre+ cells (Figure 1K), demonstrating that the experimental approach was not effective in reducing HIF-1α protein in cardiac fibroblasts. The robust induction of GFP protein confirmed Tcf21-driven iCre expression and tamoxifen-induced recombination in a subset of cells. However, this was insufficient to diminish *Hif1a* mRNA (Figure 1L) or HIF-1α protein.

To exclude potential issues with the recombination capacity of the mTmG and *Hif1a* loci, we transduced cFb_cult_ from Tcf21-iCre- mice with adenoviral constructs expressing Cre recombinase or β-galactosidase as a control. Cre transduction caused all cells to switch from red to green fluorescence, confirming the integrity and recombination competency of the mTmG locus (Figure 1M). Additionally, Cre-mediated recombination strongly reduced *Hif1a* mRNA in cFb_cult_ (Figure 1N), confirming that the *Hif1a* locus could be successfully targeted. These results suggested that in vivo only a small fraction of cardiac fibroblasts expressed iCre and at low levels, which raised the question whether Tcf21-iCre was suitable to target adult cardiac fibroblasts by tamoxifen injections.

To exclude the possibility that isolation and culture of cFb selected for a specific subtype of cFb (e.g., HIF-1α^+^ Fbs), we examined heart sections two weeks post-tamoxifen for GFP-positive fibroblasts. No GFP-positive fibroblasts (PDGFRα-positive) were detected in ventricular sections from multiple hearts (Figure 1O). Collectively, these data confirm that with our experimental approach, only a limited subset of resident cardiac fibroblasts is targeted.

### 3.2. Fibroblasts Are Not Targeted with Tcf21-iCre After TAC

**Figure 2:**
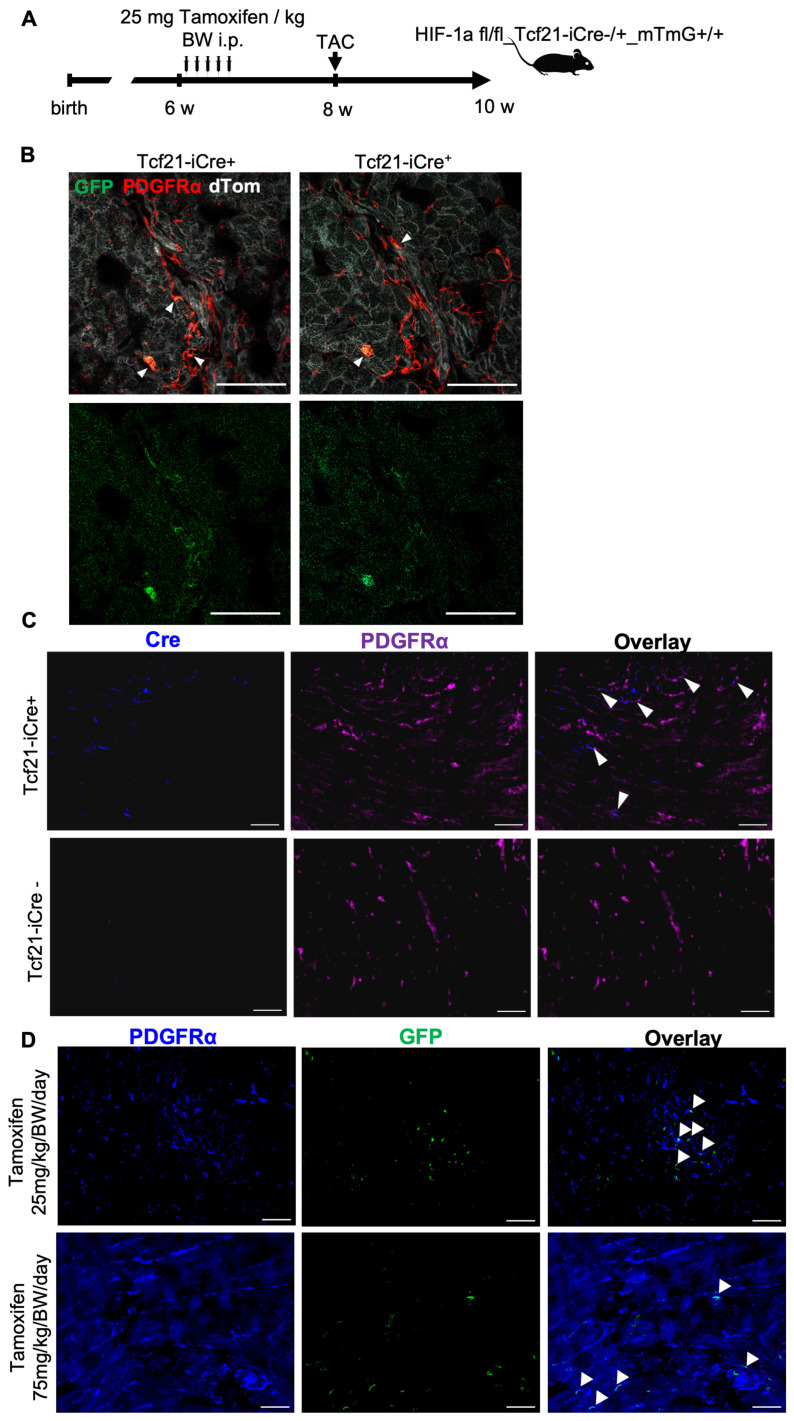

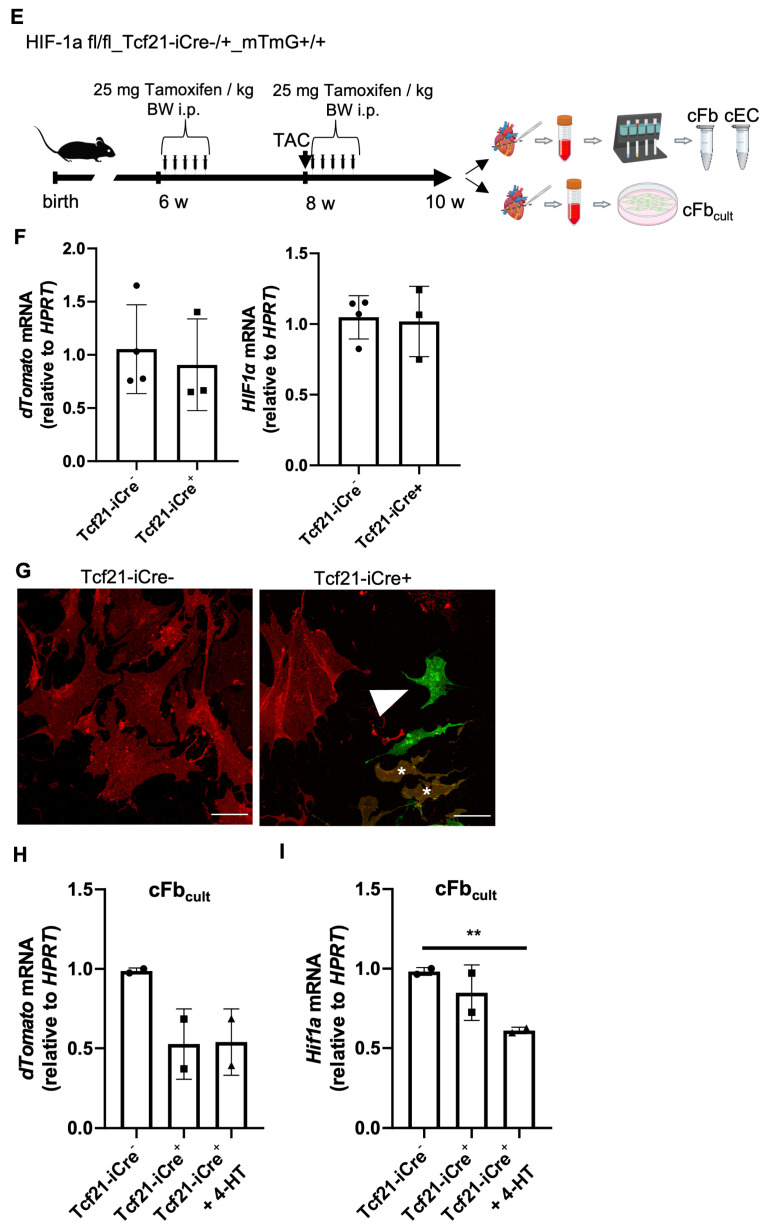

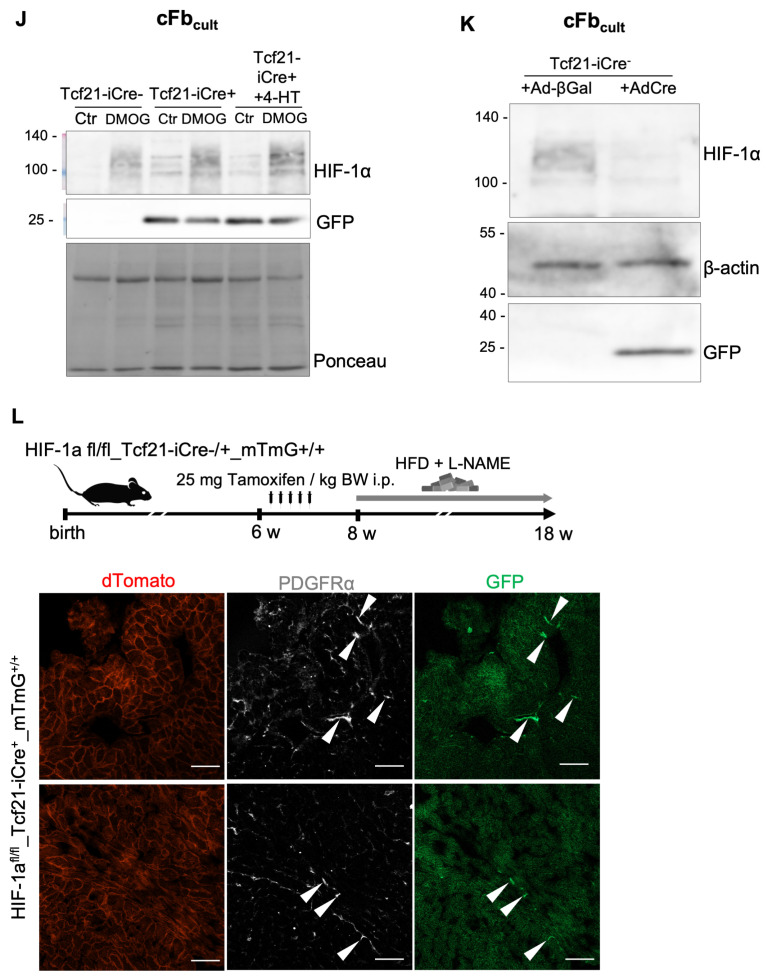
Only a small subset of resident cFb is recombined upon i.p. tamoxifen delivery in mouse models of cardiac disease. (**A**) Experimental scheme: two weeks after the tamoxifen application, transverse aortic constriction (TAC) surgery was performed, and hearts were harvested two weeks later. (**B**) Representative immunofluorescence image of two individual samples from heart tissue from Tcf21-iCre+ mice two weeks after TAC; fibroblasts were stained with an antibody against PDGFRα (red). The GFP signal appears in green. Arrowheads point to GFP/PDGFRα double-positive cells. (**C**) Tcf21-iCre- and Tcf21-iCre+ mice were treated with tamoxifen (5 d i.p., 25 mg/kg BW) and two weeks later, hearts were extracted, embedded, sectioned and stained for PDGFRα and Cre (scale bar: 50 μm). Arrowheads indicate cells positively stained for both Cre and PDGFRα. (**D**) Tcf21-iCre+ mT/mG mice were treated with 75 or 25 mg/kg bodyweight tamoxifen/day (5 d i.p.), and four hours later, hearts were extracted, embedded, sectioned and stained for PDGFRα and endogenous GFP (scale bar: 50 μm). Arrowheads point to GFP/PDGFRα double-positive cells. (**E**) Experimental scheme: two weeks after the tamoxifen application (25 mg/kg BW/day), transverse aortic constriction (TAC) surgery was performed, tamoxifen delivery was repeated for 5 d i.p. (25 mg/kg BW/day), and two weeks later, ventricular fibroblasts were isolated via MACS and then analyzed by qPCR or isolated and cultured. (**F**) *dTomato* and *Hif1a* mRNA were measured via qPCR in MACS-sorted cFb from Tcf21-iCre- and Tcf21-iCre+ mice. (**G**) cFb were cultured, fixed and imaged (scale bar: 50 μm). Arrowhead points to a GFP-positive cell. * Indicates a dTomato/GFP double-positive cell. (**H**) *dTomato* mRNA was measured via qPCR in lungs, TAC hearts and Sham hearts from Tcf21-iCre+ and Tcf21-iCre- mice. (**I**) *HIF1a* mRNA was measured via qPCR in lungs, TAC hearts and Sham hearts from Tcf21-iCre+ and Tcf21-iCre- mice; ** *p* ≤ 0.01. (**J**) cFb_cult_ were incubated with 1 mM DMOG or vehicle for 4 h, lysed, separated by SDS-PAGE and immunoblotted with anti-HIF-1α, anti-GFP and anti-β-actin antibodies. For each data point, ventricles of at least three individual mice with the same genotype were pooled. (**K**) cFb_cult_ from Tcf21-iCre- mice were transduced with adenoviruses expressing β-Gal or Cre recombinase, and 24 h later, they were incubated with 1 mM DMOG for 4 h, lysed, separated by SDS-PAGE and immunoblotted with anti-HIF-1α, anti-GFP and anti-β-actin antibodies. For each data point, ventricles of at least three individual mice with the same genotype were pooled. (**L**) Experimental design and representative immunofluorescence images of heart tissue from Tcf21-iCre+ mice stained for PDGFRα. The GFP signal appears in green. Arrowheads point to GFP/PDGFRα double-positive cells.

Cardiac fibroblasts constitute only approximately 11% of all cells in a healthy ventricular myocardium [20,21], which may explain why we could not detect recombined cFb in tissues from healthy hearts. Because the fibroblast population expands after pressure overload, we hypothesized that GFP-labeled cFb would be more readily detectable two weeks after transverse aortic constriction (TAC) surgery (Figure 2A). After induction of fibroblast expansion by TAC, only a small proportion of PDGFRα-positive cellsshowed green fluorescence (Figure 2B, white arrowheads), confirming our previous finding that most fibroblasts did not undergo efficient DNA recombination in response to tamoxifen application in Tcf21-iCre+ animals.

We performed a co-staining for Cre-recombinase and PDGFRα in hearts after TAC surgery but found that only a small subset of PDGFRα+ cells also expressed Cre, suggesting low Cre transcription at the *Tcf21* locus (Figure 2C). Accordingly, endogenous *Tcf21* mRNA was significantly lower expressed in adult mouse hearts after sham or TAC surgery compared to lung tissue (Supplemental Figure S1A,B). We also detected Cre staining in cells that were not labeled by PDGFRα (Figure 2C).

To exclude insufficient tamoxifen dosing as a reason for low recombination efficiency, we injected a three times higher amount of tamoxifen i.p. in Tcf21-iCre+ mice over a 5-day course (75 mg/kg BW/tamoxifen/day) and compared this to Tcf21-iCre+ mice injected with the lower tamoxifen dose used in the other experiments in our study (25 mg/kg BW/tamoxifen/day). Four hours after the last tamoxifen injection, the mice were sacrificed and analyzed for GFP staining (indicating successful recombination) in conjunction with PDGFRα labeling. Despite the higher dose, we identified only a rather limited number of PDGFRα-expressing fibroblasts positive for GFP (Figure 2D, white arrowheads) in the high-tamoxifen-dose group, which did not appear to be higher compared to the lower-dose group. Because tamoxifen should induce a (partial) nuclear translocation of the Cre recombinase, we investigated its cellular localization by immunostaining of the myocardium. As shown in the Supplemental Figure S1C, we detected only a small degree of nuclear Cre localization, which did not differ between mice receiving 25 mg/kg BW/day versus 75 mg/kg BW/day tamoxifen. These results indicated that target locus recombination by Tcf21-iCre is limited to a small subset of fibroblasts and that an increase in tamoxifen dosing in the 5-day injection scheme does not improve recombination efficiency at the mTmG locus.

Although not revealed by our data (Supplemental Figure S1A), previous work by Braitsch et al. suggested that *Tcf21* was induced in cFb in response to pressure overload [13]. Therefore, we added a second 5-day course of tamoxifen injections starting on the day of TAC surgery to enhance targeting efficiency (Figure 2E).

Despite this, no measurable reduction in dTomato or *Hif1a* mRNA expression was observed in cFb from Tcf21-iCre+ animals (Figure 2F), indicating that the additional tamoxifen administration did not lead to a significant targeting of the cardiac fibroblast population.

To get further insights into the recombination efficiency, we isolated and cultured cFb_cult_ two weeks after TAC with double tamoxifen administration (in total 50 mg/kg bodyweight tamoxifen). While cFb_cult_ from Tcf21-iCre− mice showed exclusively red fluorescence, a subset of cFb_cult_ from Tcf21-iCre+ mice expressed GFP (Figure 2G). Some cells displayed both red and green fluorescence, reflecting recombination of one allele again (Figure 2G, *). Consistent with these results, we measured a modest reduction in *dTomato* mRNA in cFb_cult_ from Tcf21-iCre+ mice (Figure 2H), whereas *Hif1a* mRNA expression remained unchanged compared to controls (Figure 2I). Due to limitation of the material, we unfortunately were not able to assess DNA recombination in these cells. Similarly, HIF-1α protein levels were not reduced in cFb_cult_ from Tcf21-iCre+ animals following exposure to the hypoxia mimetic DMOG for 4 h (Figure 2J), demonstrating that this approach is insufficient to decrease HIF-1α protein levels in most cardiac fibroblasts.

To exclude the possibility that the low recombination rate resulted from poor in vivo tamoxifen metabolism, cFb_cult_ from Tcf21-iCre+ mice were exposed to metabolically active 4-hydroxy-tamoxifen (4-HT) for 3 days. This treatment only minimally decreased *Hif1a* mRNA, caused no further reduction in dTomato mRNA and did not reduce HIF-1α protein under pseudo-hypoxic conditions (Figure 2J).

To confirm that recombination of the *Hif1a* locus was achievable in principle, Cre recombinase was delivered via adenoviral transduction to cFb_cult_ from Tcf21-iCre− mice. This resulted in a pronounced reduction in HIF-1α protein under pseudo-hypoxic conditions (Figure 2K), confirming that the loxP sites were intact and recombination of this genomic locus was possible.

Taken together, this suggested that the low recombination rate in most cardiac fibroblasts was due to low iCre expression levels rather than problems with tamoxifen availability or with the genomic loci. We hypothesize that the Tcf21 promoter was only active in a subset of cardiac fibroblasts in our experimental approach, and therefore, Cre expression and tranlocation was limited to selected cells as seen by the rare nuclear Cre recombinase signal.

### 3.3. Fibroblasts Are Not Targeted with Tcf21-iCre in a HFpEF Model

Heart failure with preserved ejection fraction (HFpEF) has recently emerged as a major focus in cardiovascular research. In mice, HFpEF can be induced by combining a high-fat diet (HFD) with the endothelial nitric oxide synthase inhibitor L-NAME in the drinking water [19]. Because cardiac fibrosis represents a hallmark of HFpEF, fibroblast-specific gene targeting may provide a promising therapeutic approach. However, the experimental design complicates tamoxifen administration via chow, making intraperitoneal (i.p.) delivery the method of choice.

Tcf21-iCre+ and Tcf21-iCre mice received tamoxifen i.p. for five consecutive days at six weeks of age at 25 mg/kg BW/day to activate Cre-mediated recombination at the mTmG and the *Hif1a* loci. Two weeks later, animals underwent 10 weeks of HFD + L-NAME treatment (Figure 2L).

In heart sections, only a small fraction of PDGFRα-positive cells exhibited green fluorescence (Figure 2L). This indicates that only a very small subset of resident cardiac fibroblasts can be targeted in this common HFpEF mouse model by Tcf21-iCre.

## 4. Discussion

We show here that the specific targeting of adult cardiac fibroblasts with Tcf21-iCre (inducible Cre recombinase under the promoter of the endogenous *Tcf21* gene) and the administration of tamoxifen via intraperitoneal injections for 5 days at a dose of 25 mg/kg bodyweight/day or 75 mg/kg bodyweight/day in mice does not lead to efficient recombination of floxed gene(s) in most fibroblasts during homeostatic conditions, after TAC and upon high-fat diet and L-Name administration. Hence, we have detected successful recombination of the targeted gene(s) only in a small subset of cardiac fibroblasts and show proof of Cre-mediated recombination of only one allele in multiple cells. The latter indicates that tamoxifen had successfully reached those cells but that the *Tcf21* promoter exerted an insufficient activity to drive Cre expression.

We therefore suspect that the *Tcf21* promoter had a very low activity, and we raise the question whether it is the right promoter to target most adult cardiac fibroblasts for research studies. We also observed locus-specific recombination efficiency: the mTmG locus was more readily recombined than the *Hif1a* locus as shown by qPCR from isolated fibroblasts. Adding the metabolically active 4-hydroxytamoxifen to isolated fibroblasts minimally improved the recombination of the *Hif1a* locus, which had not responded to tamoxifen in vivo before, while recombination of the mTmG locus did not further improve versus tamoxifen, indicating that different loci require different nuclear Cre levels for efficient recombination.

While all resident cardiac fibroblasts in the adult mouse heart arise from progenitor cells that were positive for Tcf21 during the postnatal period [12], different studies have come to different conclusions about the expression of *Tcf21* in adult cardiac fibroblasts. Several investigations have clearly established that adult cardiac cells with an active *Tcf21*-promoter are positive for cardiac fibroblast markers like Col1a1, PDGFRα, and Vimentin [12,14,20]. Furthermore, Tcf21-positive cardiac fibroblasts were shown to be the primary source of (Periostin-producing) myofibroblasts [14], which justifies the approach to target Tcf21-positive cells to study fibrosis. In these studies, cardiac fibroblasts were identified as Tcf21+ via a fluorescent reporter inserted into the ROSA26 locus, which was activated by Tcf21-iCre-dependent recombination. With this approach, cells with an active *Tcf21* promoter at the time of tamoxifen administration were fluorescently labeled, regardless of whether one or both alleles of the locus were recombined [12,14,20].

Several other studies show evidence that *Tcf21* is not expressed in most adult fibroblasts in the healthy heart. Using Tcf21-iCre to delete *Pdgfra* from cardiac fibroblasts, Ivey et al. noticed that only 50% of cardiac murine fibroblasts (PDGFRα^+^) were targeted and that many cells still expressed PDGFRα, which prompted the suspicion that only one allele had been successfully recombined [22]. Xiao et al. performed a cluster analysis after Tcf21-iCre-mediated deletion of Lats1/2 and showed that only two out of five clusters of cardiac fibroblasts had been targeted [23]. Another study by Tsai et al. found that fewer than 40% of all PDGFRα+ cardiac fibroblasts were also Tcf21+ [24]. Johansen et al. reached 30% more adult cardiac fibroblasts when they employed a PDGFRα-iCre rather than the Tcf21-iCre [25]. They also demonstrated that the deletion of Tcf21 from adult cardiac fibroblasts did not have effects on fibroblast numbers, myofibroblast differentiation, or fibrosis after different heart injury protocols [25]. The lack of effects of Tcf21 deletion could indicate that adult cardiac fibroblasts have extremely low expression levels of the *Tcf21* gene, which would explain why Tcf21-iCre might not be the most efficient tool for targeting adult cardiac fibroblasts. On the other hand, some studies have successfully used the Tcf21-iCre model in mice to specifically delete a gene of interest from adult cardiac murine fibroblasts. Xiang et al. successfully depleted β-catenin from a subset of adult cardiac fibroblasts, as seen by a reduction in β-catenin protein levels in cells that were Tcf21+ based on a fluorescent reporter [26]. Khalil et al. found that upon Tcf21-iCre-mediated deletion of Hsp47 (reduction was not shown on the mRNA or protein level), the mouse heart was protected against fibrosis after TAC [27]. Deleting Lats1/2 with Tcf21-iCre from a subset of cardiac fibroblasts led to increased fibrosis [23]. Using the Tcf21-iCre promoter, Francisco et al. achieved a reduction in YAP protein in the non-cardiomyocyte fraction of isolated cells, which led to an attenuation of fibrosis in response to pro-fibrotic stimuli [28]. Liu et al. showed a Tcf21-iCre-mediated reduction in p53 protein levels in IHC of heart sections post-TAC that was accompanied by an increased fibrotic response [29]. Taken together, these reports show that it is possible to successfully delete a gene of interest from a (sub)population of cardiac fibroblasts using Tcf21-iCre and to affect fibrosis and cardiac function.

While in some of these studies, very similar tamoxifen administration schemes to the one in our study were used (i.p. at 5 days consecutive days), in others, tamoxifen was delivered via chow for 1 or 2 weeks before injury or additionally after the injury until the end of the experiment, or only after the injury until the end of the experiment. While longer tamoxifen exposure times can lead to higher rates of targeted cells in mice, it is also known to have a cardiotoxic effect that could introduce experimental changes [30,31]. Also, the delivery of tamoxifen via chow to mice is not permitted in all animal facilities and might interfere with experimental protocols that are based on specific chows, such as a high-fat diet (HFD).

There are several factors that could explain variable success across studies. First, different floxed loci showed different recombination efficacies, meaning some target genes might achieve sufficient deletion to produce phenotypic effects, while others remain largely intact despite identical Cre-driver and tamoxifen protocols. In addition, different mouse backgrounds with different metabolization of tamoxifen can lead to different effective doses of the active metabolites, and recombination efficiency might differ because of different gene accessibility. The biological properties of the target genes also matter: if the gene to be deleted is a very strong driver of fibrosis, even a small reduction in that gene product in a small subset of murine cardiac fibroblasts could have significant effects. If the gene product has only a very small anti-fibrotic effect, its reduction might not lead to any measurable effect.

Additionally, if the targeted gene has a very high transcription level and can easily compensate for the loss of one allele on the protein level—like HIF-1α—Tcf21-iCre might not be an adequate tool for this model in mice. In this case, PDGFRα-iCre could be more suitable; however, we strongly recommend confirming the successful reduction in the protein of interest and adapting the experimental model and tamoxifen administration schemes accordingly.

In conclusion, we demonstrate here that the Tcf21-iCre exerts very low gene-recombination efficiencies in cardiac fibroblasts following intraperitoneal tamoxifen administration in adult mice under homeostatic conditions as well as after TAC and during high-fat diet/L-Name administration. As the most likely reason, we identified low Cre expression levels from the *Tcf21* gene locus that were limited only to a few selected fibroblasts, reducing overall recombination efficiency.

## Data Availability

The original contributions presented in this study are included in thearticle/Supplementary Materials. Further inquiries can be directed to the corresponding author.

## Supplementary Materials

The following supporting information can be downloaded at https://www.mdpi.com/article/10.3390/cells15151326/s1, Figure S1: Expression of endogenous Tcf21 and cellular localization of iCre after tamoxifen induction title.

## Abbreviations

The following abbreviations are used in this manuscript:

TAC	Transverse aortic constriction
cFb	Cardiac fibroblast
cEC	Cardiac endothelial cell
cFb_cult_	Cultured cardiac fibroblast
MACS	Magnetic activated cell sorting
iCre	Inducible Cre-recombinase
HFD	High-fat diet
L-NAME	N(ω)-nitro-L-arginine methyl ester
ECM	Extracellular matrix
FCS	Foetal calf serum
HFpEF	Heart failure with preserved ejection fraction
HFrEF	Heart failure with reduced ejection fraction
Ad-Cre	Adenovirus encoding Cre recombinase
Ad-βgal	Adenovirus encoding β-galactosidase
i.p.	Intraperitoneal
p.o.	Per os
HPRT	Hypoxanthine-Guanine Phosphoribosyl transferase
DMOG	Dimethyloxalylglycine
4-HT	4-hydroxy-tamoxifen
