# Supplementary material for: Reassessing Tcf21-iCre for Adult Cardiac Fibroblast Gene Deletion: Limited Recombination Efficiency Following Tamoxifen Injections
Source: Cells. 2026 Jul 24;15(15):1326. doi: 10.3390/cells15151326 (PMC13464824; doi:10.3390/cells15151326)
Supplement: Supplementary file 1 [file cells-15-01326-s001.zip › cells-4246102-supplementary.pdf]

Supplemental Figure

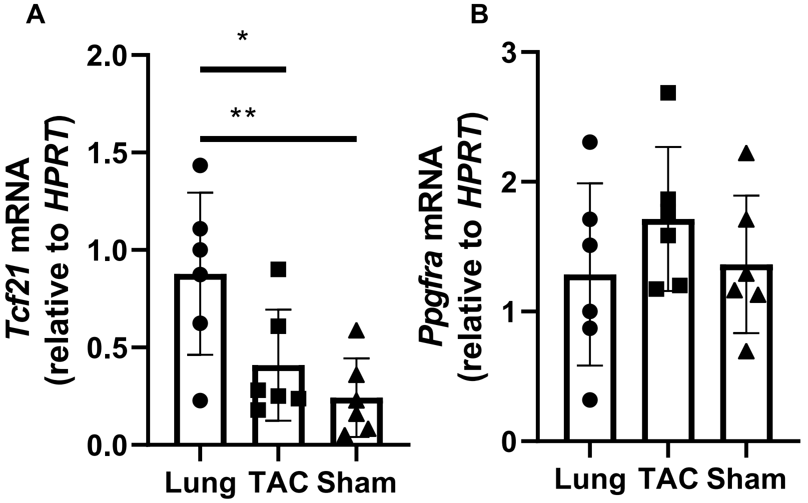

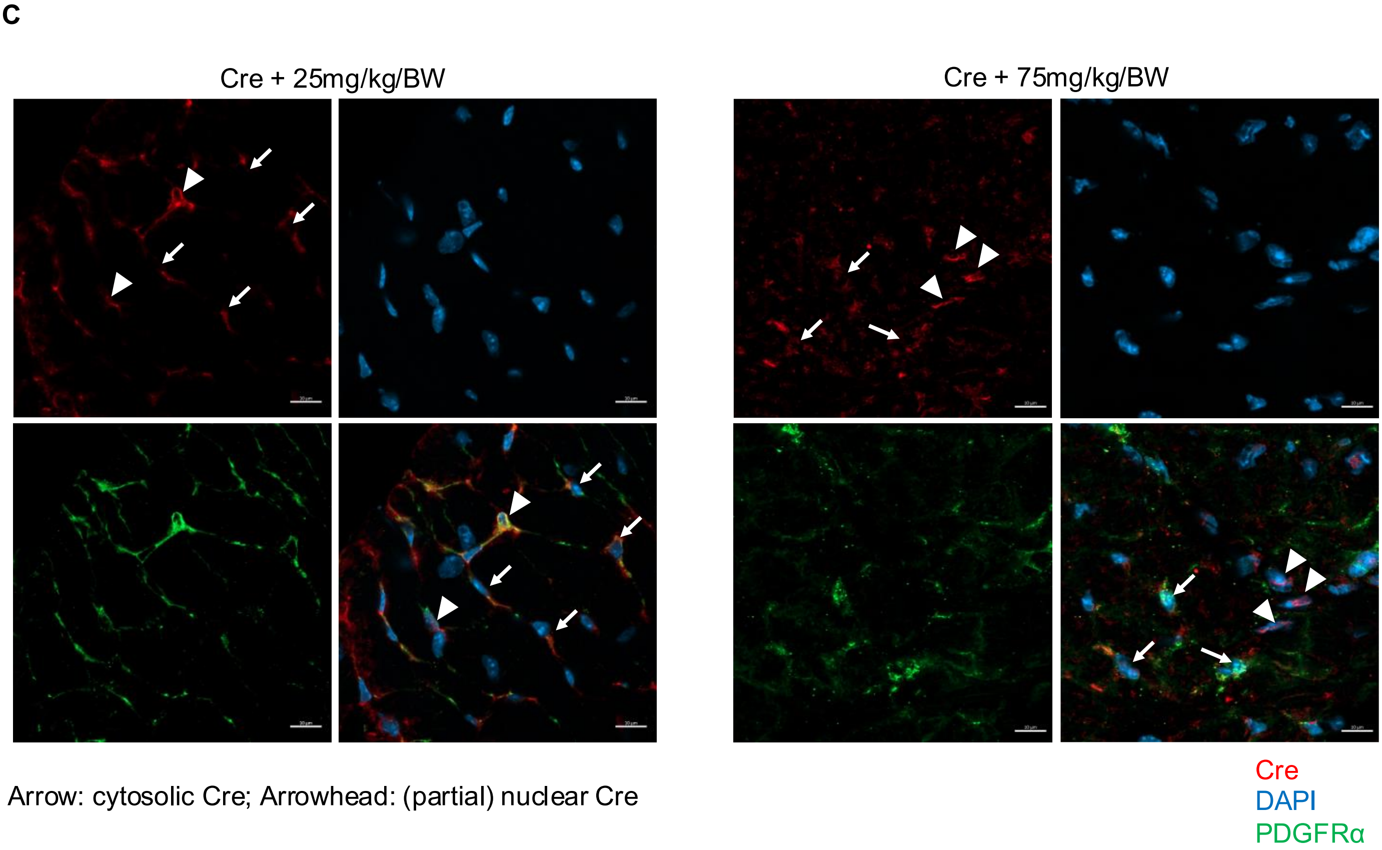

Arrow: cytosolic Cre; Arrowhead: (partial) nuclear Cre
